# The expression and role of serotonin receptor 5HTR2A in canine osteoblasts and an osteosarcoma cell line

**DOI:** 10.1186/1746-6148-9-251

**Published:** 2013-12-12

**Authors:** Shay Bracha, Austin Viall, Cheri Goodall, Bernadette Stang, Craig Ruaux, Bernard Seguin, Patrick E Chappell

**Affiliations:** 1 Department of Clinical Sciences, Oregon State University, College of Veterinary Medicine, 172 Magruder Hall, Corvallis, OR 97331-4802, USA; 2 Department of Biomedical Sciences, Oregon State University, College of Veterinary Medicine, 172 Magruder Hall, Corvallis, OR 97331-4802, USA; 3 CSU Flint Animal Cancer Center, Veterinary Teaching Hospital, Campus delivery 1678, 300 West Drake Road, Fort Collins, CO 80523, USA

**Keywords:** Osteosarcoma, Serotonin, 5HTR2A, Ritanserin

## Abstract

**Background:**

The significance of the serotonergic system in bone physiology and, more specifically, the importance of the five hydroxytryptamine receptor 2A (5HTR2A) in normal osteoblast proliferation have been previously described; however the role of serotonin in osteosarcoma remains unclear. Particularly, the expression and function of 5HTR2A in canine osteosarcoma has not yet been studied, thus we sought to determine if this indoleamine modulates cellular proliferation in vitro. Using real time quantitative reverse transcription PCR and immunoblot analyses, we explored receptor expression and signaling differences between non-neoplastic canine osteoblasts (CnOb) and an osteosarcoma cell line (COS). To elucidate specific serotonergic signaling pathways triggered by 5HTR2A, we performed immunoblots for ERK and CREB. Finally, we compared cell viability and the induction of apoptosis in the presence 5HTR2A agonists and antagonists.

**Results:**

5HTR2A was overexpressed in the malignant cell line in comparison to normal cells. In CnOb cells, ERK phosphorylation (ERK-P) decreased in response to both serotonin and a specific 5HTR2A antagonist, ritanserin. In contrast, ERK-P abundance increased in COS cells following either treatment. While endogenous CREB was undetectable in CnOb, CREB was observed constitutively in COS, with expression and exhibited increased CREB phosphorylation following escalating concentrations of ritanserin. To determine the influence of 5HTR2A signaling on cell viability we challenged cells with ritanserin and serotonin. Our findings confirmed that serotonin treatment promoted cell viability in malignant cells but not in normal osteoblasts. Conversely, ritanserin reduced cell viability in both the normal and osteosarcoma cells. Further, ritanserin induced apoptosis in COS at the same concentrations associated with decreased cell viability.

**Conclusions:**

These findings confirm the existence of a functional 5HTR2A in a canine osteosarcoma cell line. Results indicate that intracellular second messenger signal coupling of 5HTR2A is different between normal and malignant cells, warranting further research to investigate its potential as a novel therapeutic target for canine osteosarcoma.

## Background

Osteosarcoma is the most common skeletal tumor in dogs, sharing many characteristics with the disease in people. Surgery and adjuvant chemotherapy are the standard of care; however, poor outcomes often result due to pulmonary metastasis in the progressive stage of the disease. There is a pressing need for a better understanding of the pathophysiology of osteosarcoma, and the development of novel therapeutic targets for this disease.

An increasing body of evidence suggests an important role for five hydroxytryptamine (5HT) in bone physiology [1-4]. Osteoblasts, osteocytes, and osteoclasts express functional five hydroxytryptamine receptor (5HTR), and several functions exhibit a dependency on circulating 5HT [5,6]. Furthermore, a correlation between chronic administration of 5HT reuptake inhibitors with loss of bone mineral density and development of osteoporosis has been described [1,7].

Five-hydroxytryptamine, commonly known as serotonin, is produced by two enzymatic steps from the amino acid tryptophan, reactions that occur in the cytoplasm [8]. The rate-limiting enzyme is tryptophan hydroxylase, which is expressed primarily in the gut and the central nervous system [9,10]. Enterochromaffin cells, located in the gastrointestinal tract, are responsible for producing and secreting the majority of circulating serotonin. This serotonin is sequestered by platelets, which are the primary storage site in the circulation [11]. The physiological roles of systemic serotonin include regulation of intestinal motility, platelet aggregation, and regulation of blood pressure [12-14]. A second reservoir of serotonin, produced in the central nervous system, is independent of systemic storage and exhibits a different overall function [15]. The 5HT receptor (5HTR) superfamily is comprised of seven receptor types, most of which are G-coupled proteins [16,17]. These transmembrane proteins have a ligand-binding domain at the extracellular amino-terminus and an intracellular carboxy-terminus which activates the intracellular subunits [16,17].

Serotonin receptor type 2A (5HTR2A) is involved in a variety of physiological functions, including tracheal smooth muscle contraction and aldosterone production. The expression of 5HTR2A has previously been demonstrated on osteoblasts [18]. Further examination of this receptor has shown a modulatory role in cell proliferation, directly regulating bone remodeling [3,5]. Exposure of 5HTR2A to highly selective agonists activates downstream signaling via the ERK pathway, mediating osteoblastic proliferation and differentiation. The use of an antagonist on anaplastic osteoblasts resulted in decreased cell viability [3].

The regulation of bone remodeling by serotonin receptors, and more specifically the role of 5HTR2A in osteoblast proliferation, suggest that this receptor has potential as a novel therapeutic target for treatment of canine osteosarcoma. To date, the expression patterns and role of 5HTR2A in canine osteosarcoma have not been described. In the current study, we evaluated the expression and functionality of 5HTR2A in normal canine osteoblasts and an osteosarcoma cell line, and discuss its potential as a novel therapeutic target.

## Methods

### Cell lines

COS is a well characterized immortal canine osteosarcoma cell line [19]. CnOb, non-malignant canine osteoblasts, were purchased from Cell Application Inc. (San Diego, CA). COS was grown in light-treated, serum-free RPMI-1640 media and supplemented with L-glutamine, sodium pyruvate, HEPES buffer and penicillin/streptomycin. CnOb was grown in proprietary media supplied by the manufacturer. MCF7 is a human breast cancer cell line, grown in 5% serum supplemented RPMI-1640 media and supplemented with L-glutamine, sodium pyruvate, HEPES buffer and penicillin/streptomycin. Cells were incubated at 37°C in an atmosphere containing 5% CO [2].

### PCR and sequencing

Cells were harvested in 1 mL Trizol (Invitrogen, Carlsbad, CA). RNA was extracted following manufacturer’s instructions and reprecipitated in the presence of glycogen to increase purity. Quantity and purity of RNA was assessed on a Nanodrop-1000 spectrophotometer (Thermo Scientific, Wilmington, DE). One microgram RNA was converted to cDNA using High Capacity RNA to cDNA Reverse Transcription Kit (Applied Biosystems, Carlsbad, CA) per manufacturer’s instructions. The PCR mix contained 0.2 mM dNTP, 1.5 mM MgCl_2_, 1x PCR buffer, 0.4 μM primer mix (Table 1), and 1.0 U Taq Polymerase in a total volume of 50 μL. Reactions were subjected to an initial step of 94°C for 2 min, followed by 40 cycles of 94°C for 30 sec, 60°C for 30 sec, 72°C for 1 min and a final extension step of 72°C for 2 min. Amplicons were electrophoresed on a 1.5% agarose gel, and the visualized bands were cut and purified with the QIAquick gel extraction kit (Qiagen, Valencia, CA). Sequencing was performed by the Center for Genome Research and Biocomputing at Oregon State University. Sequences were compared against the published canine *htr2a* sequence (NC_006604.3 and NM_001005869.1) at the National Center of Biotechnology Information website.

**Table 1:** **Primers used for PCR of *htr2a***

**Primers**	**Location**	**Expected product size**
F1 GCTTCCGTGTGACAGAGACA	−103	632 bp
R2 CCCAGCAGCATATCAGCTA	529	
F3 CGGTCGTGATTATTCTGACCA	245	818 bp
R4 CCCTGTGGATTGATCGTTG	1062	
F5 GGGCTACAGGATGATTCCAA	640	836 bp
R6 CCCCCCAGATAGGTGAAAA	1475	

### Real-time quantitative reverse transcription PCR (real-time qRT-PCR)

The total reaction volume was 10 μL and contained 1.7 μL H_2_O, 5 μL Power SYBR (Invitrogen), 0.8 μL primer mix and 2.5 μL cDNA (pre-diluted 6-fold in water). Real-time qRT-PCR was run in triplicate on a MicroAmp® Optical 96-well reaction plate in a Step One Plus unit (Applied Biosystems). The protocol included a 10 min initiation step at 95°C, followed by 40 cycles of 95°C for 5 sec, 59°C for 30 sec, and 70°C for 35 sec. Relative *htr2a* expression was calculated by the 2^−ΔΔCT^ method in relation to their endogenous expression of *ywhaz* and to CnOb [20,21]. The housekeeping gene *ywhaz* was chosen based on previously published data, and also since no difference was found in mean CT between COS *ywhaz* and CnOb *ywhaz* (mean Ct value +/− 95% CI for mean) [20,21]. Primers were designed to flank a large intron so that amplification of residual genomic DNA would be avoided: *ywhaz* 5′-AGCCTGCTCTCTTGCAAAGAC-3′ and 5′-GGGTATCCGATGTCCACAATG-3′, *htr2a* 5′-CCCATTCTTCATCACGAACAT-3′ and 5′-GGAGAGGTAACCGATCCAGAC-3′. Experiments were replicated three times for both CnOb and COS.

### Immunoblot

Confluent COS cell cultures were treated for 24 hours in media without FBS and then challenged with escalating concentrations of serotonin (Tocris) and ritanserin (Tocris), a specific 5HTR2A antagonist. The same protocol was followed for CnOb, excluding the starvation phase which could not take place due to the use of the commercial supplied media. Serotonin and ritanersin were dissolved in DMSO according to manufacturer’s instructions. The challenges were: serotonin (3.125, 12.5, 50 μM), ritanserin (3.125, 2.5, 50 μM), concurrent serotonin (12.5 μM) and ritanserin (3.125, 12.5, 50 μM), and vehicle carrier (DMSO). Cells were harvested after a 10 min exposure by washing twice with 1 × PBS, lysing in 500 μL RIPA buffer containing phosphatase and proteinase inhibitors, and centrifugation at 8,300 × g for 5 min. Protein concentrations were determined with the Pierce® BCA protein assay. Homogenate aliquots (containing 20 μg protein) were mixed at a volume ratio of 4:1 with 10 mM Tris–HCl buffer (pH 6.8) containing 10% glycerol, 2% SDS, 0.01% bromophenol blue and 5% β-mercaptoethanol, boiled for 10 min at 100°C, and electrophoresed on a SDS–PAGE gel at a constant current of 10 mA/plate. Separated proteins from COS, CnOb and MCF7 (positive control) were transferred to a nitrocellulose membrane, blocked for 45 min with 1% BSA dissolved in TBST [20 mM Tris–HCl (pH 7.4) containing 150 mM NaCl, and 0.1% Tween-20], followed by overnight incubation at 4°C with anti-5HTR2A goat antibody (Santa Cruz sc-32538) diluted 1:200 in blocking buffer [22]. Proteins labeled with primary antibody were detected with donkey anti-goat secondary antibody conjugated to IR800 and scanned on an Odyssey Imager (Li-Cor, Lincoln, NE). The membrane was stripped and re-probed with anti-α-tubulin rabbit antibody (Santa Cruz sc-12462-R) diluted 1:1000 in block buffer, and detected with a goat anti-rabbit secondary antibody. A mouse monoclonal phosphorylated ERK antibody (Santa Cruz sc-7383), followed by secondary anti-mouse HRP-conjugated antibody (Santa Cruz sc-2005), was used to probe the same extracts. This membrane was stripped and incubated again with a primary ERK antibody (Santa Cruz sc-93) and detected by goat anti-rabbit secondary antibody (Santa Cruz sc-2004). This membrane was stripped for the third time and incubated with anti-α tubulin antibody (Santa Cruz sc-12462-R) detected by goat anti-rabbit secondary antibody (Santa Cruz sc-2004). Similarly, phosphorylated CREB (Cell Signaling 9198) and CREB (Santa Cruz sc-186) antibodies were used to probe new membranes and detected with goat anti-rabbit secondary antibody. Membranes were developed with Supersignal West Pico Chemiluminescent Substrate (Thermo Scientific) on radiography film. A-431 Whole Cell Lysate (Santa Cruz) was used as positive control for the ERK and P-ERK, while SK-N-MC (Cell Signaling) was used as a positive control for CREB and CREB-P.

### Cell viability assays

Proliferation assays were performed using COS and CnOb cell lines; these experiments were replicated three times, with each experiment using three replicates for each sample. Proliferation was estimated using the CellTiter 96® Aqueous One Solution (Promega), which is a MTS tetrazolium assay. Cells were plated in 96-well culture plates at 20,000 cells per well and both cell lines incubated in light-treated media for 24 hours, followed by 24 hours of serum free media for COS, after which time the media was replaced by a fresh media containing serotonin, ritanserin, serotonin and ritanserin, or DMSO as a vehicle control. Each cell line was challenged with increasing concentrations of serotonin or ritanserin, ranging from 0-50 μM, for 24 hours. DMSO concentration (0.1%) remained constant for all samples [3,23]. In addition, malignant cells were challenged with serotonin (12.5 μM) and escalating concentrations of ritanserin (0-50 μM). Cells were processed following manufacturer’s instructions and absorbance was measured at 550 nm on a microplate reader. Relative viability values were calculated using the formula: Viability Index = (mean absorbance of treated cells)/(mean absorbance of control cells).

### Apoptosis assay

Induction of apoptosis was evaluated by quantification of caspase 3 and caspase 7 activities in treated and untreated COS only. Caspase activity was determined by a commercially available luminogenic caspase-3/7 substrate assay (Caspase-Glo® 3/7 Assay, ProMega). Briefly, cells were seeded in 96-well plates at a density of 10,000 cells/well with serum-free RPMI media and permitted to adhere for 24 hours. This medium was replaced and cells were challenged with fresh, serum-free RPMI media containing the following drugs at the specified concentrations: serotonin (3.125, 6.25, 12.5, 25, 50 μM), ritanserin (3.125, 6.25, 12.5, 25, 50 μM), and concurrent serotonin (12.5 μM) and ritanserin (3.125, 6.25, 12.5, 25, 50 μM). In addition, cells were pre-incubated with the general caspase inhibitor Z-VAD-FMK (CalBiochem) at 25 μM for 60 min before being challenged with fresh RPMI containing ritanserin at 12.5 μM. For a positive control, cells were incubated with taurolidine (TauroPharm GmbH) at 125.0 μM, which has been previously demonstrated to induce apoptosis in COS [24]. As a negative control, cells were incubated with the vehicle carrier DMSO. Cells were challenged for 12 hours and then the caspase activity assay was performed according to manufacturer specifications. Cumulative luminescence over a 1 sec interval was measured in a GloMax® 96 Microplate Luminometer (Promega). All drug challenge and control samples were performed in triplicate.

### Statistical analyses

For the real-time qRT-PCR, the relative quantification of *htr2a* expression between COS and CnOb was evaluated with a two-tailed, unpaired T test with Welch’s correction. For the cell viability data, one-way ANOVA with Dunnett’s correction was utilized to compare the viability index at each concentration of serotonin, ritanserin, and serotonin + ritanserin to the vehicle control. One-way ANOVA with Sidak’s correction was employed to compare the proliferation index at each concentration of ritanserin to the equivalent concentration of serotonin + ritanserin. The 50% inhibitor concentration (IC50) of ritanserin for COS was calculated using non-linear regression of the log of the inhibitor versus a variable slope response equation, with constraints set at 100% for the top and 0% for baseline. A one-way ANOVA with Dunnett’s correction was used to analyze caspase activity at varying concentrations of serotonin, ritanserin, and serotonin and ritanserin, compared to the vehicle control. One-way ANOVA with Sidak’s correction was employed to assess caspase activity in response to increasing concentrations of ritanserin in comparison to the equivalent concentrations of serotonin and ritanserin. Assessment of caspase activity between ritanserin (12.5 μM) treated cells with and without pretreatment with the caspase inhibitor were compared with an unpaired Student’s T-test. Statistical significance was assigned for calculated values of *p* < 0.05. Comparisons were performed with a commercially available statistical software package (GraphPad Prism).

## Results

### PCR and real-time qRT-PCR

**Figure 1:**
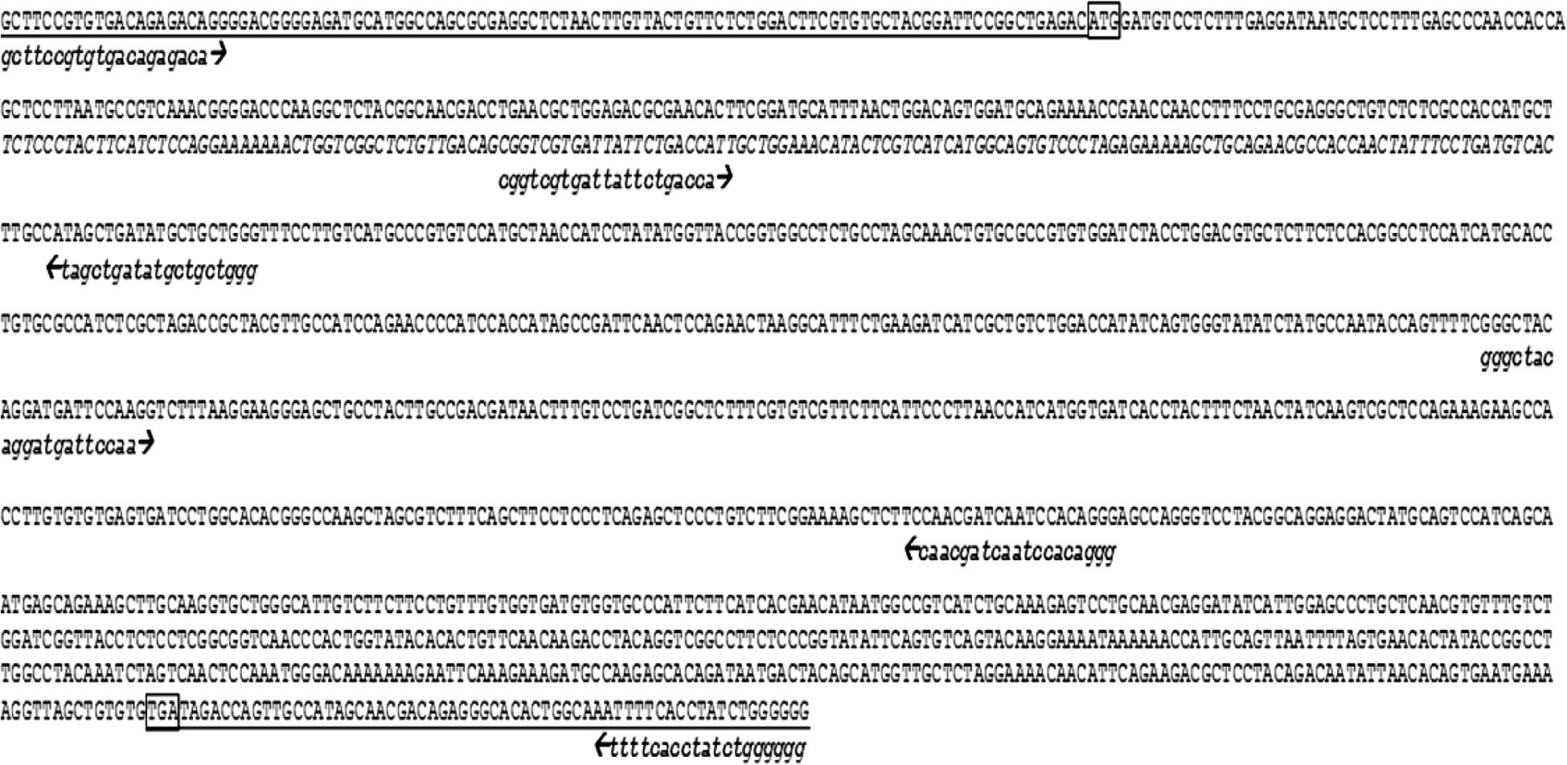
**PCR/sequencing primers mapped to reference canine *htr2a *sequence (NC_006604.3 and NM_001005869.1).** Underlined sequence indicates 5′ and 3′ UTRs. Start and Stop codons are boxed.

**Figure 2:**
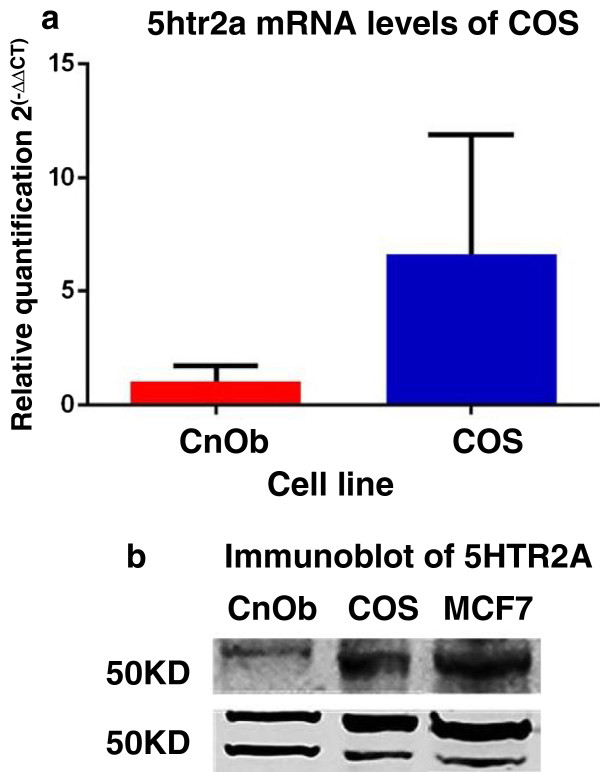
**Expression of 5HTR2A is higher in COS in comparison to CnOb. a: ***htr2a* mRNA levels of COS normalized to CnOb. The graphed bars are mean RQ +/− 95% confidence interval of mean. The mean difference in expression between the malignant cell line and CnOb is 5.577 +/−1.124 (*p* = 0.434). **b:** Immunoblot of 5HTR2A. Representative immunoblot demonstrating 5HTR2A protein abundance in COS and CnOb.

Expression of *htr2a* was confirmed by PCR, and sequencing and alignment of overlapping amplicons revealed the absence of mutations in *htr2a* in COS compared to the reference sequence from Genbank (Figure 1). When compared to the expression level of *htr2a* in CnOb, COS demonstrated a 5.6 +/−1.2 fold increase (*p* = 0.0434, Figure 2).

### Immunoblot

**Figure 3:**
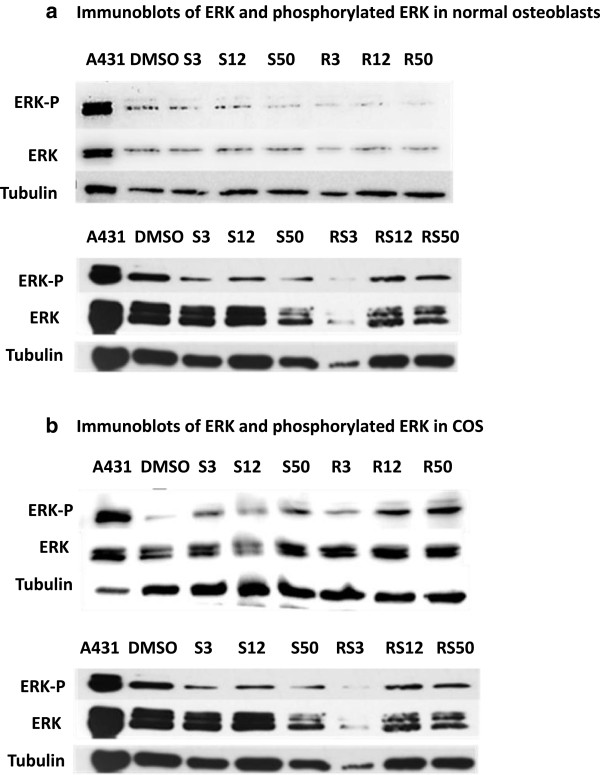
**Phosphorylation of ERK is cell line and treatment dependent. a**: Immunoblots of ERK and phosphorylated ERK in normal osteoblasts. CnOb exhibited decreased ERK phosphorylation when treated with increasing concentrations of serotonin (S), ritanserin (R) or when both drugs are combined (SR). **b**: Immunoblots of ERK and phosphorylated ERK in COS. Treatment with increasing concentrations of serotonin (S), ritanserin (R), or both drugs (SR) resulted in commensurate increases in phosphorylation.

**Figure 4:**
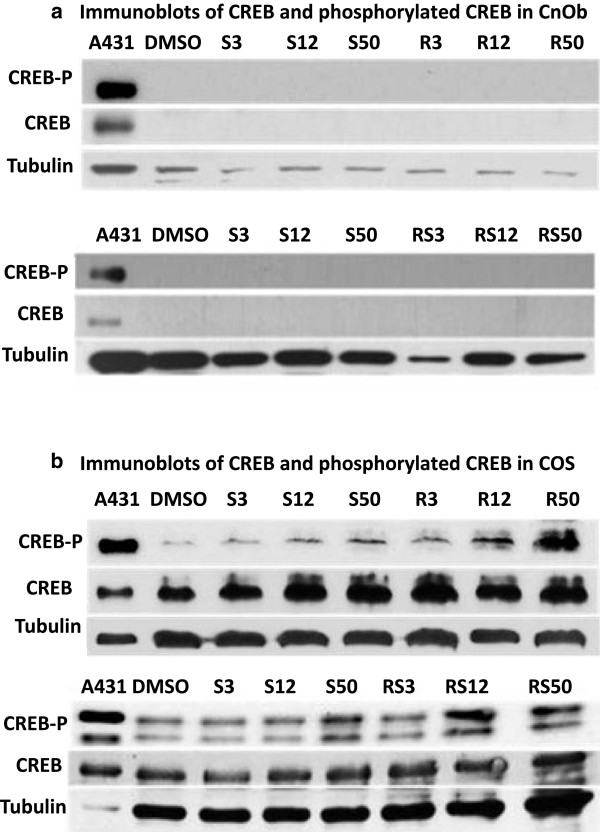
**Phosphorylation of CREB by ritanserin is evident in COS but not in CnOb. a**: Immunoblots of CREB and phosphorylated CREB in CnOb. Immunoblot of CnOb confirming the absence of endogenous CREB expression. No treatments resulted in an induction of CREB. **b**: Immunoblots of CREB and phosphorylated CREB in COS. CREB phosphorylation increases at high concentrations of serotonin and intermediate to high concentrations of ritanserin, or with a combination of the two drugs.

After probing with anti-5HTR2A, a 50 KDa size band was visible in the protein lysates of CnOb, COS and MCF7 (Figure 1c), consistent with previous reports of 5HTR2A in MCF7 cell extracts (Figure 1c) [22]. ERK was constitutively present and phosphorylated in CnOb when treated with vehicle. CnOb cells challenged with escalating doses of serotonin, ritanserin, or concurrent serotonin and ritanserin, exhibited a slight reduction of phosphorylation in comparison to control (Figure 3a). Conversely, increases in ERK-P above control levels were observed in COS incubated with serotonin, ritanserin, or serotonin and ritanserin (Figure 3b). Endogenous CREB was not evident in CnOb (Figure 4a), yet this transcription factor, in both unphosphorylated and phosphorylated forms, was constitutively found in COS lysates (Figure 4b). While serotonin did not increase phosphorylation of CREB when applied to COS, ritanserin induced phosphorylation in a dose-dependent manner (Figure 4b).

### Cell viability assays

**Figure 5:**
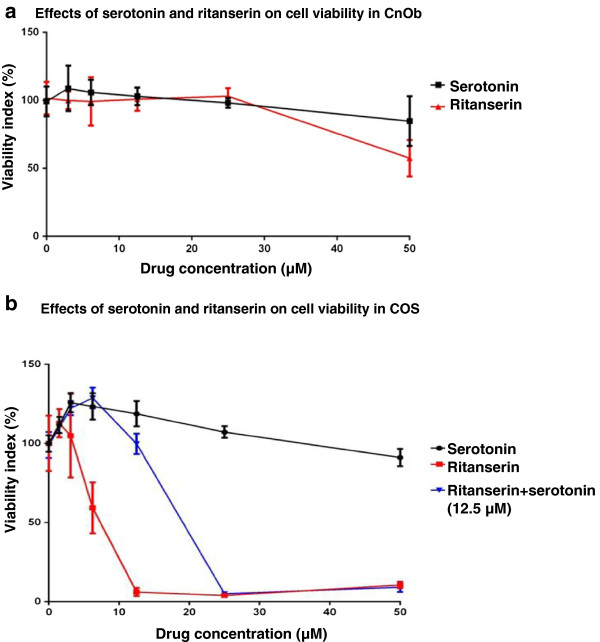
**Effect of serotonin and ritanserin on cell viability is cell line dependent. a:** Effects of serotonin and ritanserin on cell viability in CnOb. Cell viability was assessed through a MTS assay of CnOb treated with escalating doses of serotonin or ritanserin. Decreased cell viability relative to the vehicle control is only observed at 50 μM serotonin (*p* = 0.013) and 50 μM ritanserin (*p* < 0.01). A greater decrease in viability is observed at 50 μM ritanserin than 50 μM serotonin (*p* < 0.01). Plotted values are mean cell viability +/− 95% confidence interval of mean. **b**: Effects of serotonin and ritanserin on cell viability in COS. Cell viability was assessed through a MTS assay of COS treated with escalating doses of serotonin, ritanserin, and ritanserin with concurrent serotonin (12.5 μM). Relative to the vehicle control, increased viability was observed at 3.125, 6.25, and 12.5 μM serotonin (*p* < 0.01). Conversely, a mild decrease in viability was observed at 6.25 μM ritanserin (*p* < 0.01) with marked decreased evident at 12.5, 25.0, and 50 μM (*p* < 0.01). Viability was greater at 6.25 and 12.5 μM ritanserin when concurrently incubated with serotonin (12.5 μM) than the viability observed with 6.25 and 12.5 μM ritanserin alone (*p* < 0.01). The calculated IC50 of ritanserin for COS is 6.3 μM. Plotted values are mean cell viability +/− 95% confidence interval of mean.

Treatment with escalating concentrations of serotonin promoted increased viability relative to the vehicle control in COS at 3.125, 6.25, and 12.5 μM (*p* < 0.01) (Figure 5). Conversely, cell viability returned to levels equivalent to non-treated cells at 25.0 and 50.0 μM. Following ritanserin treatment, a moderate decrease in COS viability was found at 6.25 μM (*p* < 0.01), with marked decreases observed at 12.5, 25.0, and 50.0 μM (*p* < 0.01) (Figure 5). The calculated IC50 for ritanserin treatment of COS cells was 6.3 μM. When concurrently incubated with 12.5 μM serotonin, a higher viability index was observed at 6.25 μM and 12.5 μM ritanserin than with similar doses of ritanserin alone (*p* < 0.01) (Figure 5). No difference in cell viability was noted in CnOb cells between control and serotonin treatments except for a statistically significant decrease at 50 μM (*p* = 0.013). Moderate decreases in viability were observed in CnOb cells treated with at 50 μM ritanserin (*p* < 0.01). The decreased viability observed with 50 μM ritanserin was greater than the viability decrease with 50 μM serotonin (*p* < 0.01).

### Apoptosis assay

**Figure 6:**
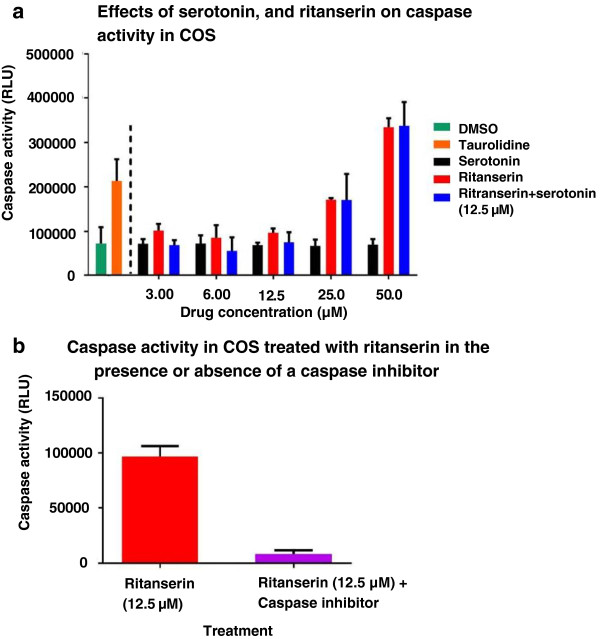
**Ritanserin induce caspase activity in COS. a**: Effects of serotonin, and ritanserin on caspase activity in COS. Caspase activity increased with escalating concentrations of ritanserin, with mildly increased activity appreciated at 3.125 (*p* = 0.0170) and 12.5 μM (*p* = 0.025) and markedly increased activity present with 25.0 and 50.0 μM (*p* < 0.01). No difference in caspase activity between the DMSO vehicle control and any concentration of serotonin were observed. Concurrent incubation of ritanserin with 12.5 μM serotonin showed attenuated caspase activity at 3.125 (*p* = 0.049) and 6.25 μM ( *p* = 0.043) ritanserin relative to cells treated with ritanserin alone. Caspase activity in taurolidine treated cells, the positive control, was significantly greater than the vehicle control (*p* < 0.01). Plotted values are mean caspase activity +/− 95% confidence interval of mean. **b**: Caspase activity in COS treated with ritanserin in the presence or absence of a caspase inhibitor. Pre-incubation with the general capase inhibitor Z-VAD-FMK dramatically decreased caspase activity in COS cells treated with 12.5 μM ritanserin (*p* < 0.01). Plotted values are mean caspase activity +/− 95% confidence interval of mean.

Relative to vehicle control, COS cells exhibited mild increases in caspase activity with 3.125 μM (*p* = 0.017) and 12.5 μM (*p* = 0.025) ritanserin, and marked increases in activity with higher doses (25.0 and 50.0 μM, *p* < 0.01) (Figure 6a). No increase in caspase activity was observed at any concentration of serotonin (Figure 6a). Cells concurrently incubated with 12.5 μM serotonin expressed lower caspase activity at 3.125 μM (*p* = 0.049) and 6.25 μM (*p* = 0.043) ritanserin than when treated with 3.125 and 6.25 μM ritanserin alone (Figure 6a). However, no difference in caspase activity was observed between 12.5, 25.0, and 50.0 μM ritanserin with serotonin (12.5 μM) versus 12.5, 25.0 and 50.0 μM ritanserin alone. Pre-incubation with the caspase inhibitor dramatically reduced caspase activity at 12.5 μM ritanserin (Figure 6b).

## Discussion and conclusion

Systemic 5HT plays an important role in normal bone remodeling by acting on osteoblasts via three receptor isoforms: 5HTR1B, 5HTR2B, and 5HTR2A [2,3,6]. The predominance of 5HTR2A in premature osteoblasts promotes osteogenesis [13]. However, while 5HTR2A expression has been demonstrated in anaplastic osteoblasts, there is limited information about the expression and function of the serotonergic system in canine osteosarcoma cells, and whether this circulating signal performs similar roles in malignant versus normal cells. The 5HTR2B receptor is expressed only in differentiated mature cells and regulates proliferation [4]. In contrast to 5HTR2A, 5HTR1B activation exerts a direct inhibitory effect on the expression and phosphorylation of CREB and thus cyclin D activity, consequently halting osteoblastic proliferation upon agonist binding [25]. This has been demonstrated in an *in vivo* model exploring the role of 5HTR1B and its dependency on circulating 5HT, where osteoporosis was observed in 5HTR1B knockdown mice [25].

In the current study, we investigated the role of 5HTR2A on intracellular signaling and cellular proliferation in the canine osteosarcoma cell line COS. We found increased *htr2a* expression in COS compared to canine osteoblasts, an increase correlated with higher levels of 5HTR2A protein. Previous reports have shown expression of *htr2a* from early phases of the proliferative period throughout maturation in anaplastic osteoblasts, confirming a proliferative role of activated 5HTR2A in these cells [10,22]. Our data are in agreement with these results, demonstrating increased cell viability of COS following serotonin treatment. Interestingly, the effect of 5HT is confined to malignant cells, as CnOb exhibited no increases in cell viability at any serotonin concentration. Further, while the highly specific 5HTR2A antagonist ritanserin significantly decreased COS cell viability at comparatively low concentrations, CnOb viability was unchanged at concentrations below 50 μM. In COS, this decreased viability appears to be secondary, at least in part, to induction of apoptosis rather than direct cell cytotoxic effects. These findings demonstrate a selective effect of ritanserin on the malignant cell line, while sparing the normal osteoblasts, highlighting the potential of 5HTR2A as an attractive therapeutic target for osteosarcoma.

While phosphorylated ERK was observed in both CnOb and COS in this study, the relative phosphorylation of ERK was augmented with increasing doses of both serotonin and ritanserin only in the malignant cell line. In contrast, CnOb demonstrated decreased phosphorylation following treatment with these drugs. Being a nonspecific agonist, serotonin may activate 5HTR2A, 5HTR1B and 5HTR2B, resulting a net effect similar to that of the antagonist. This discrepancy suggests that the traditional receptor signaling via 5HTR2A is altered in neoplastic cells. Interestingly, changes in the phosphorylation status of ERK did not correlate with cell viability of either COS or CnOb thus the role of ERK signaling in the serotonergic system in canine osteosarcoma remains unclear. However, in COS, higher doses of ritanserin correlated with increased CREB phosphorylation and decreased cell viability. Accordingly, caspase activity was found to reciprocate increased ritanserin doses. In normal murine osteoblasts, phosphorylation of CREB has been previously described to play a crucial part in apoptosis, with phospho-CREB reported to induce cell proliferation while its suppression through 5HTR1B activation resulted in decreased cell viability [25]. However, induction of apoptosis through phospho-CREB has been observed in neuronal and endothelial tissues and with CREB over-expression [26-28]. Based upon our findings, CREB abundance and phosphorylation status in neoplastic canine osteoblasts appears to be associated with apoptosis, in contrast to what is observed in normal osteoblasts. Interestingly, CREB was not detectible in CnOb, highlighting possible interspecies differences when compared to murine osteoblasts.

In conclusion, this study revealed overexpression of *htr2a* in canine osteosarcoma cells and aberrant 5HTR2A signaling compared to normal osteoblasts. In COS, 5HTR2A antagonism induced CREB phosphorylation, a directional downstream signaling pathway that is not evident in normal osteoblasts. CREB phosphorylation status correlated with cell viability *in vitro*, with low doses of the 5HTR2A inhibitor selectively decreasing osteosarcoma cell viability, while having no significant effect on normal osteoblasts. This attenuated viability appears to be through induction of apoptosis. Together, these results suggest that pharmacological inhibition of 5HTR2A may represent a potential novel target in osteosarcoma therapy due to differential expression and altered signal coupling found in this cancer. Future studies targeting 5HTR2A *in vivo* as a novel therapeutic target and exploring species-dependency of these effects are warranted.

## Competing interests

The authors declare that they have no competing interests.

## Authors’ contributions

SB was involved in study design, performed some immunoassays, carried out molecular genetic studies and drafted the manuscript. AV performed cell cultures, cell proliferation assays and some immunoassays. CG carried out molecular genetic studies, sequence alignment and assisted in manuscript construction. BS performed some molecular genetic studies and sequence alignment. CR participated in study design and performed statistical analysis. BS participated in study design and manuscript construction. PC participated in study design and coordination and manuscript creation. All authors read and approved the final manuscript.
